# Modulation of extracellular matrix/adhesion molecule expression by BRG1 is associated with increased melanoma invasiveness

**DOI:** 10.1186/1476-4598-9-280

**Published:** 2010-10-22

**Authors:** Srinivas Vinod Saladi, Bridget Keenen, Himangi G Marathe, Huiling Qi, Khew-Voon Chin, Ivana L de la Serna

**Affiliations:** 1 Department of Biochemistry and Cancer Biology, University of Toledo College of Medicine, Toledo, OH 43614, USA; 2 Department of Medicine, The University of Toledo College of Medicine, 3000 Arlington Avenue, Toledo, OH 43614, USA; 3 Center for Diabetes and Endocrine Research, The University of Toledo College of Medicine, 3000 Arlington Avenue, Toledo, OH 43614, USA

## Abstract

**Background:**

Metastatic melanoma is an aggressive malignancy that is resistant to therapy and has a poor prognosis. The progression of primary melanoma to metastatic disease is a multi-step process that requires dynamic regulation of gene expression through currently uncharacterized epigenetic mechanisms. Epigenetic regulation of gene expression often involves changes in chromatin structure that are catalyzed by chromatin remodeling enzymes. Understanding the mechanisms involved in the regulation of gene expression during metastasis is important for developing an effective strategy to treat metastatic melanoma. SWI/SNF enzymes are multisubunit complexes that contain either BRG1 or BRM as the catalytic subunit. We previously demonstrated that heterogeneous SWI/SNF complexes containing either BRG1 or BRM are epigenetic modulators that regulate important aspects of the melanoma phenotype and are required for melanoma tumorigenicity in vitro.

**Results:**

To characterize BRG1 expression during melanoma progression, we assayed expression of BRG1 in patient derived normal skin and in melanoma specimen. BRG1 mRNA levels were significantly higher in stage IV melanomas compared to stage III tumors and to normal skin. To determine the role of BRG1 in regulating the expression of genes involved in melanoma metastasis, we expressed BRG1 in a melanoma cell line that lacks BRG1 expression and examined changes in extracellular matrix and adhesion molecule expression. We found that BRG1 modulated the expression of a subset of extracellular matrix remodeling enzymes and adhesion proteins. Furthermore, BRG1 altered melanoma adhesion to different extracellular matrix components. Expression of BRG1 in melanoma cells that lack BRG1 increased invasive ability while down-regulation of BRG1 inhibited invasive ability in vitro. Activation of metalloproteinase (MMP) 2 expression greatly contributed to the BRG1 induced increase in melanoma invasiveness. We found that BRG1 is recruited to the MMP2 promoter and directly activates expression of this metastasis associated gene.

**Conclusions:**

We provide evidence that BRG1 expression increases during melanoma progression. Our study has identified BRG1 target genes that play an important role in melanoma metastasis and we show that BRG1 promotes melanoma invasive ability in vitro. These results suggest that increased BRG1 levels promote the epigenetic changes in gene expression required for melanoma metastasis to proceed.

## Background

Melanoma is an aggressive malignancy, characterized by high potential for metastasis and notoriously resistant to chemotherapeutics [1,2]. The prognosis for patients with melanoma is dependent on the stage of the disease as measured by tumor thickness, ulceration, and the presence of metastases [3]. According to the American Joint Committee on Cancer staging system, Stage I melanomas are less than 1 mm thick and localized to the skin. Stage II melanomas are greater than 1 mm thick, may be ulcerated, but are still localized to the skin. In stage III, the tumor has spread to nearby lymph nodes but not yet detected at distant sites. In stage IV, the tumor has spread beyond the original area of skin and nearby lymph nodes to other organs, or to distant areas of the skin or lymph nodes. The five year survival rate for stage I, II, III, and IV is estimated to be 92%, 68%, 45%, and 11% respectively [4]. The high mortality rate associated with metastatic melanoma and the lack of effective treatment underscores the necessity to understand the mechanisms that promote melanoma progression.

The progression from a primary tumor to metastatic melanoma is a multistep process that involves detachment from the primary tumor mass, invasion into the dermis, migration through the extracellular matrix (ECM), and vasculature and colonization of distant sites [5,6]. Each of these steps involves cytoskeletal alterations as well as changes in the tumor cell's interactions with neighboring cells and with the ECM [7]. The inherently high metastatic potential associated with melanoma has been attributed to the migratory nature of neural crest derived precursors that give rise to the melanocyte lineage [8]. Metastatic potential is also dependent on pro-metastatic genetic changes such as those involving NEDD9 amplification as well as epigenetic changes that modulate the expression of genes required for each step in the process [9,10]. Thus, the propensity for melanoma to metastasize may be intrinsically determined, permanently fixed by genetic alterations, and dynamically modulated at an epigenetic level by signals from the changing microenvironment.

Epigenetic regulation of gene expression often involves changes in chromatin structure that are catalyzed by chromatin remodeling enzymes [11,12]. Two classes of enzymes remodel chromatin structure by catalyzing covalent histone modifications or by hydrolyzing ATP to mobilize nucleosomes [13]. SWI/SNF complexes are ATP dependent chromatin remodeling enzymes that have been shown to increase DNA accessibility, allowing gene specific regulators or general transcription factors to bind and to activate or repress gene expression [13]. SWI/SNF enzymes play critical roles during organism development [14]. Particularly relevant to melanoma is the regulatory role that SWI/SNF enzymes play in promoting neural crest migration and differentiation as well as SWI/SNF interactions with Microphthalmia -Associated Transcription Factor (MITF), a lineage survival oncogene in melanoma [15-17].

Mammalian SWI/SNF complexes are composed of the BRG1 or BRM catalytic ATPase subunit and 9-12 BRG1/BRM associated factors (BAFs) [18]. Diverse SWI/SNF complexes are distinguished by the particular ATPase and the presence of unique BAFs [19]. The BRG1 and BRM containing complexes have similar chromatin remodeling activity in vitro but do not necessarily have redundant functional roles *in vivo *[20]. Dependent on the cellular context, BRG1 and BRM play overlapping or distinct roles in tumorigenesis. Both BRG1 and BRM expression is down-regulated in lung cancer [21]. However, low expression of BRM has been associated with gastric cancer while high expression of BRG1 has been associated with advanced stages of gastric and prostate cancer [22-24].

Reconstitution of SWI/SNF subunits into cancer cells that lack expression typically induces a change in morphology [25,26]. Furthermore, disruption of SWI/SNF activity by the introduction of dominant negative BRG1 and BRM into normal cells dramatically alters cell size and shape and invasiveness [27]. These morphological changes parallel changes in the expression of cytoskeletal regulators, cell surface proteins, adhesion molecules, and enzymes that degrade the ECM [26-30]. Thus, SWI/SNF enzymes play an important role in regulating the expression of genes important for tumor metastasis. We previously demonstrated that BRG1 and BRM expression is variable in melanoma cell lines, such that some cell lines express elevated levels of BRG1 and BRM and a subset of cell lines are deficient in BRG1 or BRM [31]. We found that reconstitution of BRG1 in a BRG1 deficient melanoma cell line promoted expression of MITF target genes that regulate melanogenesis and survival. Furthermore, BRG1 promoted resistance to cisplatin and down-regulation of BRG1/BRM significantly compromised tumorigenicity. An independent study determined that sequential down-regulation of BRG1 and BRM inhibits melanoma proliferation [32]. These studies suggest that SWI/SNF enzymes are important epigenetic modulators of melanoma tumorigenicity and potentially regulate metastatic potential.

To further characterize BRG1 expression in melanoma, we assayed expression of BRG1 in patient derived metastatic melanomas. We found that BRG1 mRNA levels were significantly higher in stage IV tumors compared to stage III tumors and to normal skin. Furthermore, BRG1 protein levels were elevated in highly invasive human metastatic melanoma cell lines. We expressed BRG1 in an established melanoma cell line that lacks detectable levels of BRG1 and profiled expression of extracellular matrix and adhesion molecules. We found that BRG1 modulated the expression of a subset of cell surface receptors, adhesion proteins, and extracellular matrix remodeling enzymes. Furthermore, BRG1 altered adhesion to different ECM components and promoted invasion through matrigel. Activation of matrix metalloproteinase (MMP) 2 expression in BRG1 expressing cells was determined to contribute to the BRG1 mediated increase in invasive ability. Down-regulation of BRG1 in a highly invasive melanoma cell line resulted in decreased MMP2 expression and decreased invasive ability. We investigated the mechanisms involved in BRG1 mediated activation of MMP2 expression and found that BRG1 interacts with a transcriptional regulator of MMP2, the SP1 transcription factor, and is recruited to the matrix metalloproteinase (MMP) 2 promoter. In combination, these results suggest that BRG1 plays a role in promoting melanoma progression by regulating the expression of metastasis associated genes.

## Results

### BRG1 is highly expressed in metastatic melanoma

**Figure 1:**
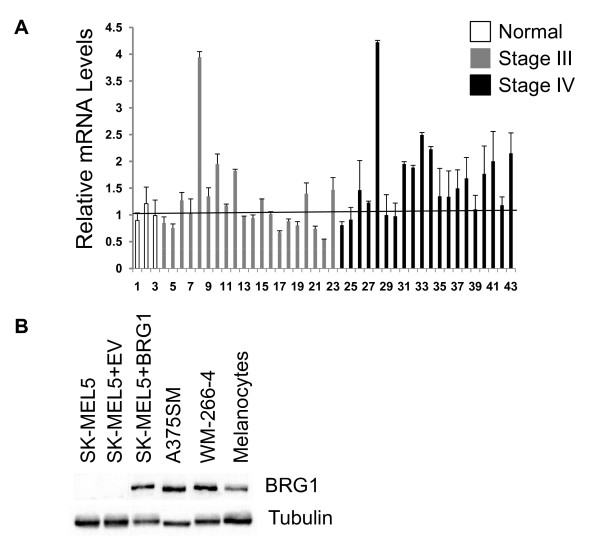
**BRG1 is highly expressed in patient derived melanomas and metastatic melanoma cell lines**. A. Tissue Scan Melanoma qPCR Arrays (Origene) containing cDNAs from patient derived normal skin, stage III melanomas, and stage IV melanomas were subjected to qRT-PCR with BRG1 specific primers. BRG1 levels were normalized to β-actin. The results were averaged from values obtained by performing three PCR arrays. B. Western blot comparing BRG1 levels in two invasive melanoma cell lines (A375SM and WM-266-4) and human epidermal melanocytes with that in BRG1 deficient SK-MEL5 cells, SK-MEL5 cells reconstituted with empty vector (EV), and BRG1 reconstituted SK-MEL5 cells. Total cell lysate was loaded. Tubulin is a loading control.

To evaluate BRG1 expression during melanoma progression, we examined BRG1 mRNA levels using quantitative (qPCR) arrays (Origene) containing normalized cDNA prepared from patient derived normal skin (3 samples), from stage III (21 samples) and stage IV (19 samples) metastatic melanoma specimen. Although BRG1 mRNA levels were lower in a subset of individual melanoma samples compared to normal skin, the average level of BRG1 was higher in stage III (1.2 fold) and stage IV melanoma (1.7 fold) compared to that in normal skin (Figure. 1A). The higher levels of BRG1 in stage IV melanoma compared to normal skin was statistically significant (p < .05). There was also a statistically significant increase in BRG1 mRNA levels in stage IV melanoma compared to stage III melanoma (p < .01). Although there was also a trend toward increased BRG1 expression in stage III melanoma compared to normal skin, the increase was not statistically significant, possibly due to an insufficient normal skin sample size. Interestingly, microarray profiling of primary melanoma tumors compared to normal skin revealed that BRG1 mRNA levels in primary melanoma is significantly higher than in normal skin [33,34] (Additional file. 1). In combination, these data suggest that BRG1 mRNA levels are elevated in primary melanoma compared to normal skin and increase during disease progression (from stage III to IV).

We and others determined that SK-MEL5 cells, derived from an axillary node melanoma, are deficient in BRG1 expression [31,32]. To determine whether BRG1 protein levels are consistently down regulated in other metastatic melanoma cell lines, we compared BRG1 protein levels in SK-MEL5 cells with levels in two highly metastatic melanoma cell lines, A375SM and WM-266-4. The A375SM cell line was established from a lung metastasis formed by injection of parental cells into nude mice [35]. The WM-266-4 cell line was derived from a lymph node metastasis [36]. We found that both A375SM and WM-266-4 express high levels of BRG1 compared to SK-MEL5 cells and to normal human melanocytes (Figure. 1B). We previously reported that re-introduction of BRG1 in SK-MEL5 cells promotes pigmentation as well as increased resistance to cisplatin [31]. As shown in Figure. 1B, BRG1 reconstituted SK-MEL5 cells express BRG1 at similar levels as A375SM and WM-266-4, which we previously estimated to be approximately 2 fold higher than that in normal melanocytes [31].

### BRG1 modulates extracellular matrix and adhesion molecule expression in SK-MEL5 melanoma cells

A previous microarray study showed that re-expression of BRG1 in a BRG1/BRM deficient human adrenal adenocarcinoma cell line (SW13 cells), activated the expression of 80 genes and repressed the expression of 2 genes [28]. Many of the BRG1 regulated genes were cell surface proteins and extracellular matrix remodeling enzymes or secreted proteins such as CD44, E-cadherin, matrix metalloproteinase (MMP) 2, and osteonectin (SPARC) [28-30]. Thus, re-expression of BRG1 in BRG1/BRM deficient adenocarcinoma cells alters the expression of a subset of genes, and in particular the expression of genes that potentially have important roles in regulating tumor metastasis.

**Figure 2:**
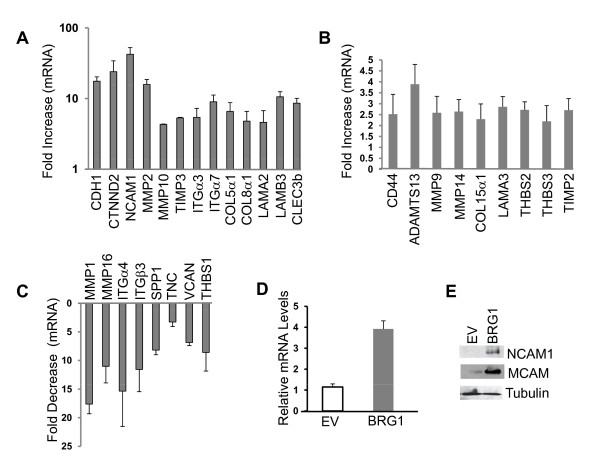
**BRG1 modulates the expression of extracellular matrix and adhesion molecule expression in SK-MEL5 cells**. The expression of extracellular and adhesion molecule related genes was profiled in control SK-MEL5 (empty vector) and BRG1 reconstituted SK-MEL5 cells using a focused qPCR array. The expression level of each gene was normalized to a control value obtained by averaging the expression of four housekeeping genes. The average of three to four independent experiments is shown. All changes in gene expression are statistically significant as determined by the student's t test (p value equal to or less than 0.05). A. Expression of genes that are activated greater than four-fold by BRG1. B. Expression of genes that are activated greater than two fold by BRG1. C. Expression of genes that are down-regulated by BRG1. D. MCAM expression was evaluated separately from the qPCR array using gene specific primers. Normalization of mRNA levels was to GAPDH. E. Detection of NCAM1 and MCAM protein expression by Western blotting. Tubulin is a loading control.

To evaluate how re-expression of BRG1 in the BRG1 deficient melanoma cell line, SK-MEL5, alters the expression of metastasis associated gene expression, we examined BRG1 induced changes in gene expression using quantitative RT^2 ^Profiler PCR Arrays (SABiosciences) and assayed the expression of 84 genes related to cell-cell and cell matrix interactions (Additional file 2). We found that the expression of 13 genes on the array was highly up-regulated by BRG1 (greater than 4-fold) (Figure. 2A). The most highly up-regulated genes (greater than 10 fold) were neural cell adhesion molecule (NCAM1), E-cadherin (CDH1), catenin delta 2/neural plakophilin related armadillo protein (CTNND2), MMP2, and laminin β3 (LAMB3) (Figure. 2A). Other highly activated genes (greater than 4 fold) included MMP10, tissue specific inhibitor of metalloproteinase (TIMP) 3, integrins α3 and α7, two collagen genes, and genes encoding components of the basement membrane (Figure. 2A).

BRG1 activated the expression of 10 additional genes at least two fold, including CD44, MMP9 and MMP14 (MT1-MMP) (greater than 2 fold) (Figure. 2B). Interestingly, re-expression of BRG1 also significantly inhibited the expression of 8 genes (Figure. 2C), while the remaining 53 genes on the array were not significantly affected by the expression of BRG1 (Additional file 1, Table 1). Thus our data indicate that re-expression of BRG1 in BRG1 deficient melanoma cells affects the expression of a subset of cell surface and extracellular matrix remodeling enzymes, some of which overlap (E-cadherin, CD44, and MMP2) and some which are distinct from those reported to be modulated by reconstitution of BRG1 in BRG1/BRM deficient SW13 adenocarcinoma cells. Many of the genes we found to be modulated by BRG1 (Figure. 2A, B, and 2C) encode proteins that play a role in regulating melanoma invasiveness and metastatic potential [6,7,37].

The most highly activated gene in BRG1 reconstituted SK-MEL5 cells was NCAM1 (Figure. 2A). NCAM1 is a cell adhesion molecule (CAM) in the immunoglobulin superfamily that is expressed at the cell surface and mediates cell to cell and cell matrix interactions [38]. High expression of NCAM1 in malignant neoplasms, including melanoma, is associated with an aggressive tumor phenotype [39]. Although high levels of NCAM1 have been associated with metastatic potential, the functional role of NCAM1 in melanoma has not been demonstrated, and high levels of NCAM1 have also been detected in benign nevi [40]. Thus, the role of NCAM1 in melanoma metastasis is unclear. MCAM (MUC18), a related cell adhesion molecule is over-expressed in advanced primary and metastatic melanoma. Its expression in melanoma cell lines enhances metastatic potential in nude mice [41,42]. We found that in addition to NCAM1, BRG1 significantly increased the expression of MCAM (Figure. 2D). Thus, re-expression of BRG1 in SK-MEL5 cells activated the expression of two related cell adhesion molecules that have been implicated in promoting tumor metastasis. We verified that the changes in NCAM1 and MCAM expression also occurred at the protein level (Figure. 2E). Interestingly, increased levels of the 140KD NCAM1 isoform was detected in BRG1 expressing cells. This isoform is associated with malignant neoplasms and induction of anti-apoptotic programs [39].

### E-cadherin localization to the cell junction is compromised in BRG1 reconstituted SK-MEL5 cells

Two of the most highly activated genes in BRG1 expressing SK-MEL5 cells were E-cadherin (CDH1) and catenin delta 2/neural plakophilin related armadillo protein (CTNND2) (Figure. 2A). E-cadherin is a calcium dependent transmembrane receptor that localizes to adherens junctions and mediates cell-cell adhesion. In many cancer types, loss of E-cadherin expression coincides with acquisition of an invasive phenotype and development of metastatic disease. In normal melanocytes, E-cadherin mediates melanocyte-keratinocyte interactions and loss of E-cadherin expression or a change in its cellular distribution is associated with early phases of melanoma. Furthermore, over-expression of E-cadherin in melanoma cells reduces melanoma invasiveness [43]. Thus, expression of BRG1 in SK-MEL5 cells could potentially reduce melanoma invasiveness through up-regulation of E-cadherin. Interestingly, BRG1 also promoted expression of δ-catenin/neural plakophilin-related armadillo protein (CTNND2) (Figure. 2A), but had no effect on the expression of β-catenin or α-catenin (data not shown), two other members of armadillo/β-catenin superfamily of cell adhesion molecules. Increased expression of CTNND2 in prostate cancer has been associated with redistribution and loss of E-cadherin at the adherens junction [44].

**Figure 3:**
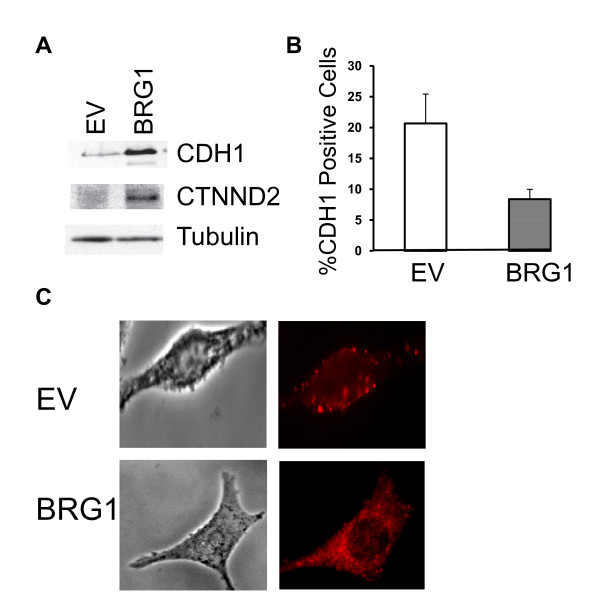
**BRG1 promotes E-cadherin expression but compromises E-cadherin localization to the cell junction in SK-MEL5 cells**. A. Detection of E-cadherin (CDH1) and CTNND2 protein expression by Western blotting. Tubulin is a loading control. B. Cells were stained with control IgG or an antibody to E-cadherin and FITC labeled secondary antibody. E-cadherin at the cell surface was quantified by FACS analysis. Significantly less E-cadherin was localized to the cell surface in SK-MEL5 +BRG1 cells compared to control SK-MEL5 cells (p < 0.05). C. E-cadherin staining of representative control SK-MEL5 cells and BRG1 expressing SK-MEL5 cells by immunofluorescence.

We verified that reconstitution of BRG1 in SK-MEL5 cells resulted in increased E-cadherin and CTNND2 expression at the protein level (Figure. 3A). To determine the status of E-cadherin at the cell surface in control SK-MEL5 cells and SK-MEL5 cells expressing BRG1, we performed flow cytometry. We found that although total E-cadherin expression increased (Figure. 3A), the localization of E-cadherin to the cell surface was reduced in cells expressing BRG1 compared to control cells (Figure. 3B). Furthermore, immunofluorescence revealed that E-cadherin was mostly cytoplasmic in BRG1 expressing SK-MEL5 cells (Figure. 3C). Reduced localization of E-cadherin to the cell surface suggested that in SK-MEL5 cells, re-expression of BRG1 may further compromise E-cadherin function.

### BRG1 alters melanoma adhesion to different ECM components

Re-expression of BRG1 in SK-MEL5 cells resulted in an altered pattern of integrin expression (Figures. 2A and 2C). Integrins are transmembrane glycoproteins that mediate specific interactions between cells and the ECM and regulate migration [45]. Hetero-dimers composed of α and β subunits serve as receptors with specificity for different ligands. Integrin expression is a key determinant of a cell's ability to attach to different ECM components and to migrate on these substrates. Aberrant integrin expression has been associated with melanoma progression [45].

**Figure 4:**
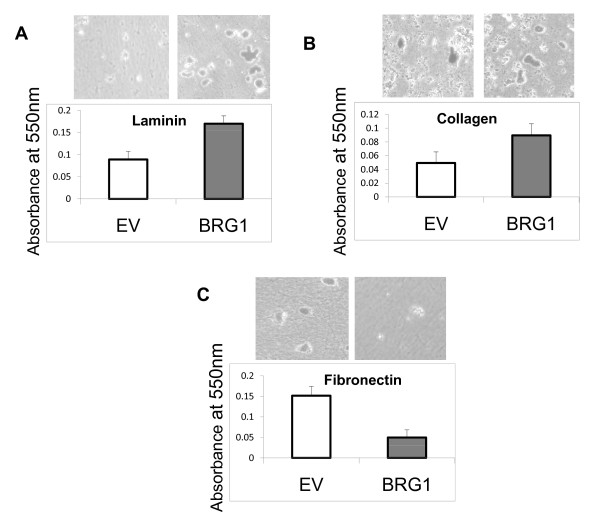
**BRG1 expression alters adhesion of SK-MEL5 cells to different ECM components**. 96 well plates were coated with laminin, collagen, or fibronectin and blocked with BSA. Control SK-MEL5 cells expressing empty vector (EV) and SK-MEL5 cells expressing BRG1 were seeded onto coated plates at a density of 2 × 10^4 ^cells. After incubation for 30 minutes, plates were washed and the cells stained with crystal violet. Control plates coated with BSA were also included but did not retain cells after washing (data not shown). Representative fields are shown (20× magnification). Adhesion was quantified by reading absorbance at 550 nm. The data shown is the average of two independent experiments done in triplicate. The fold change in adhesion to all three substrates was significantly altered by expression of BRG1 (p < 0.01). A. Adhesion to laminin. B. Adhesion to collagen. C. Adhesion to fibronectin.

BRG1 enhanced the expression of integrins α7 and α3 and inhibited the expression of integrins α4 and β3 (Figures. 2A and 2C). Modulation of integrin expression by BRG1 suggested that reconstitution of BRG1 in BRG1 deficient melanoma cells might alter melanoma cell interactions with specific ECM components. We compared the ability of the control (empty vector) SK-MEL5 cells with that of the SK-MEL5 cells expressing BRG1 to adhere to laminin, collagen, and fibronectin. We found that BRG1 expressing cells demonstrated increased adhesion to laminin and collagen and decreased adhesion to fibronectin (Figure. 4). The observed increase in adhesion to laminin is consistent with increased expression of integrin α7, which is a component of α7β1, a complex that has high affinity for laminin [46]. Increased adhesion to collagen is consistent with increased expression of α3, which is a component of the α3β1 complex that has high affinity for several ECM components, including collagen [45]. Reduced adhesion to fibronectin is consistent with decreased expression of α4, which forms the α4β1 complex and β3 which forms the αVβ3 complex, two integrins with high affinity for fibronectin [45]. The expression of these integrins is elevated in primary or metastatic melanomas [47-50], however it is not possible to designate specific integrins as "pro-neoplastic" because their effect on tumor progression is dependent on the cellular context and the specific step in tumor progression [51]. Our data indicate that BRG1 may regulate metastatic potential by modulating the integrin profile and altering adhesiveness to different ECM components.

### MMP2 activity is up-regulated in BRG1 expressing SK-MEL5 cells and contributes to increased melanoma invasiveness

**Figure 5:**
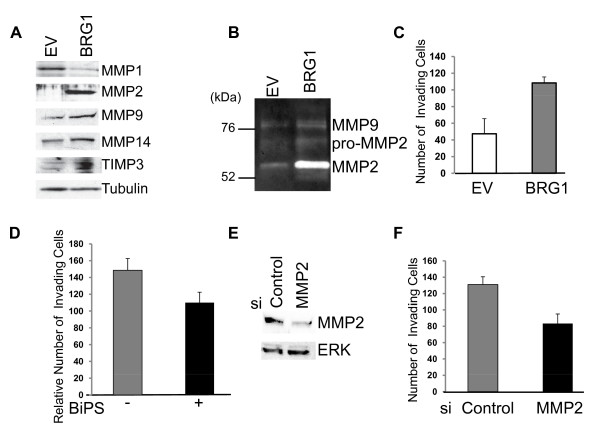
**MMP2 activity is up-regulated in BRG1 expressing SK-MEL5 cells and promotes invasion through matrigel**. A. Detection of MMP and TIMP protein expression by Western blotting. Tubulin is a loading control. B. MMP activity in control (EV) and SK-MEL5 expressing BRG1 was determined by zymography. Cells were cultured in serum free media for 24 hours and the supernatant collected and normalized to cell number. C. Invasion assays were performed using matrigel coated chambers with 5% FBS as a chemoattractant. Control SK-MEL5 cells (EV) and SK-MEL5 cells expressing BRG1 were seeded at a density of 1.25 × 10^5 ^cells per well on top of control or matrigel inserts. Percent invasion through matrigel was calculated relative to migration through the control insert. The data shown is the average of two independent experiments done in triplicate. Expression of BRG1 significantly increased invasion (p < 0.01). D. Vehicle treated (DMSO) or BiPS (10μM) treated BRG1 expressing SK-MEL5 cells were subjected to the invasion assay as described in C. The data shown is representative of two independent experiments performed in duplicate. Treatment with the MMP2/MMP9 inhibitor, BiPS significantly inhibited invasion (p < 0.01). E. SK-MEL5 +BRG1 cells were transfected with Acell SMART Pool siRNAs targeting MMP2 or red non-targeting siRNAs and analyzed by Western blotting 120 hours after transfection. F. Control and MMP2 down-regulated SK-MEL5+BRG1 cells were subjected to the invasion assay as described in C, 120 hours after transfection with siRNAs. Invasion assays were performed in triplicate. Down-regulation of MMP2 significantly compromised invasion (p < 0.01).

In addition to changes in adhesion, metastasis also requires extensive ECM remodeling. The matrix metalloproteinases (MMPs) are the main proteases that remodel the ECM [52]. Re-expression of BRG1 in SK-MEL5 cells resulted in a dramatic increase in MMP2 and MMP10 expression and a smaller but significant increase in MMP9 and MMP14 (MT-MMP1) expression (Figures. 2A and 2B) and a decrease in MMP1 and MMP16 expression (Figure. 2C). We verified that the observed changes in the mRNA profile resulted in consistent changes in protein expression for MMP1, MMP2, MMP9, and MMP14 (Figure. 5A).

Expression of MMPs is controlled at the transcriptional and post-translational levels [53]. Our data indicated that BRG1 promotes expression of MMP2, MMP9, and MMP14 at the protein level (Figure. 5A). MMP2 (gelatinase A, 72-kDa type IV collagenase) and MMP9 (gelatinase B, 92-kDa type IV collagenase are secreted as inactive pro-zymogens that are subsequently processed and activated. MMP14 (MT1-MMP) is a membrane bound MMP that activates MMP2 at the cell surface [54]. Furthermore, naturally occurring tissue inhibitor of metalloproteinases (TIMPs) down-regulate MMP activity [55]. The balance between TIMP and MMP expression is critically important in determining overall MMP activity. We found that in addition to MMPs, BRG1 also activated expression of TIMP2 and TIMP3, which would be expected to down-modulate MMP activity (Figures. 2A, 2B, and 5A).

In order to determine if re-expression of BRG1 in SK-MEL5 cells resulted in increased secretion of active MMP2 and MMP9, we performed gelatin zymography on supernatants derived from control and BRG1 expressing SK-MEL5 cells. We determined that although TIMP levels were increased, there was still a substantial increase in active MMP2 and MMP9 secreted by SK-MEL5 cells expressing BRG1 compared to BRG1 deficient SK-MEL5 cells (Figure. 5B).

The observed increase in MMP2 and MMP9 activity as well as other alterations in extracellular matrix and adhesion molecule expression suggested that BRG1 plays an important role in regulating melanoma invasiveness. To determine the overall biological consequence of BRG1 re-expression in SK-MEL5 cells, we investigated whether BRG1 promotes changes in the ability of melanoma cells to be invasive in vitro. We found that SK-MEL5 cells that express BRG1 had significantly increased ability to invade through Matrigel coated Boyden chambers (Figure. 5C).

To elucidate the mechanisms by which BRG1 promotes invasion, we treated cells with an inhibitor of MMP2/MMP9 and performed invasion assays. We found that inhibition of MMP2 and MMP9 activity partially abrogated the BRG1 mediated increase in invasive ability (Figure. 5D). Consistently, siRNA mediated down-regulation of MMP2 (Figure. 5E) also reduced the BRG1 medicated increase in invasiveness (Figure. 5F). Thus, activation of MMP2 and possibly MMP9 expression contributes to the BRG1 induced increase in SK-MEL5 invasive ability.

### Down-regulation of BRG1 in WM-266-4 cells inhibits melanoma invasiveness

**Figure 6:**
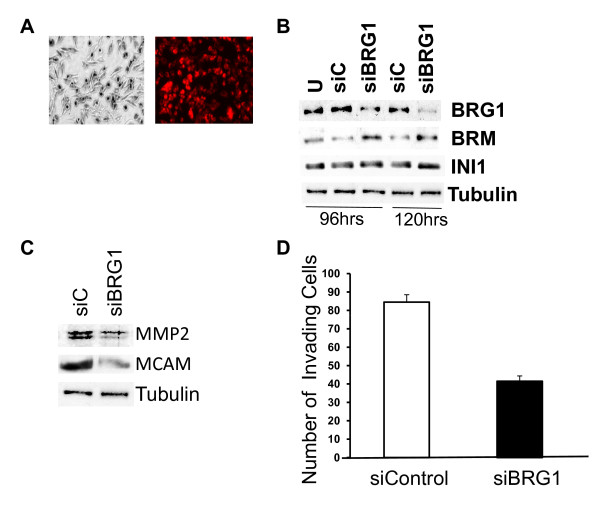
**Down-regulation of BRG1 inhibits melanoma invasion through matrigel**. A. WM-266-4 melanoma cells transfected with control (DY-547 labeled) siRNAs were visualized by phase-contrast microscopy. B. WM-266-4 melanoma cells were transfected with Acell SMART Pool siRNAs targeting BRG1 or red non-targeting siRNAs and analyzed by Western blotting. C. Control and BRG1 down-regulated WM-266-4 cells were subjected to the invasion assay as described in Figure. 5, 120 hours after transfection with siRNAs. Invasion was significantly compromised in BRG1 down-regulated cells (p < 0.01).

Most established melanoma cell lines express high levels of BRG1 [31], including two metastatic melanoma cell lines, A375SM and WM-266-4 (Figure. 1B). This raised the possibility that BRG1 is required for these cells to be invasive. To determine if loss of BRG1 compromises invasive ability in one of these highly invasive cell lines, we down-regulated BRG1 expression in WM-266-4 cells using a pool of siRNAs that target BRG1 but not the alternative ATPase, BRM (Figure. 6A and 6B). We performed a timecourse after siRNA transfection and determined that BRG1 down-regulation was effective 120 hours after transfection (Figure. 6B). Interestingly, BRM expression was slightly lower in cells transfected with control siRNA compared to untreated but then increased in BRG1 down-regulated cells. However, expression of the BRG1/BRM associated factor, INI1, did not change as a result of siRNA transfection. Previous studies have suggested that BRM expression is highly sensitive to growth conditions [56]. We found that in WM-266-4 cells, BRM expression but not BRG1 or INI1 expression is sensitive to changes in WM-266-2 confluency (Additional file 3). Thus, we speculate that siRNA transfection may have an inhibitory effect on BRM expression because of non-specific effects on proliferation. Nevertheless, BRM expression was elevated in BRG1 knockdown cells compared to both untreated cells and cells that expressed control siRNA (Figure. 6B).

We found that down-regulation of BRG1 resulted in decreased MMP2 and MCAM expression (Figure. 6C) and reduced invasion through Matrigel-coated Boyden chambers (Figure. 6D). Furthermore, although BRM levels increased in BRG1 down-regulated cells, our data suggest that BRM can not compensate for these BRG1 specific functions. Thus, both a gain of function and loss of function approach show that high levels of BRG1 promote melanoma invasive ability in vitro.

### SP1 interacts with BRG1 to regulate MMP2 expression in SK-MEL5 cells

**Figure 7:**
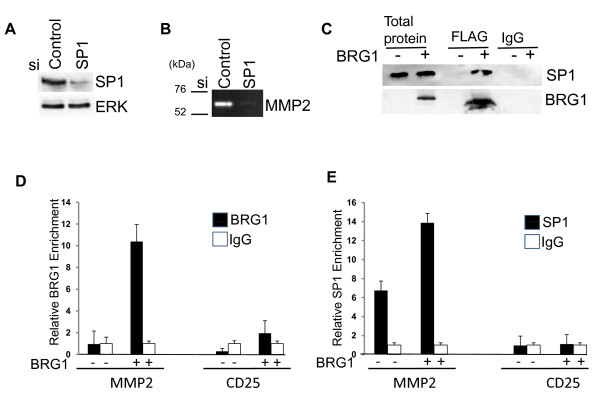
**SP1 interacts with BRG1 to regulate MMP2 expression**. A. SK-MEL5 +BRG1 cells were transfected with Acell SMART Pool siRNAs targeting SP1 or red non-targeting siRNAs. SP1 expression was analyzed by Western blotting 144 hours after transfection. B. MMP2 secretion in control and SP1 down-regulated cells was analyzed by zymography 144 hours after transfection. C. Co-immunoprecipitations were performed with FLAG antibody or control IgG. Co-immunoprecipitating proteins were analyzed by Western blotting with the indicated antibodies. D. Chromatin immunoprecipitations (ChIPs) were performed with antibodies to SP1, BRG1, or control IgG and analyzed by qPCR with primers specific for the MMP2 promoter or a control CD25 region. The results from two independent experiments were assayed twice. BRG1 was significantly enriched on the MMP2 promoter compared to the control CD25 region (p < 0.01). SP1 binding to the MMP2 promoter was significantly increased in SK+MEL5+BRG1 compared to control cells (p < 0.01).

Our data suggested that activation of MMP2 is an important mechanism by which BRG1 promotes melanoma cell invasive ability. To determine the mechanism by which BRG1 activates MMP2 expression in SK-MEL5 melanoma cells, we investigated whether BRG1 intereacts with a transcriptional regulator of MMP2. BRG1 was previously shown to directly activate the MMP2 promoter through interactions with the transcription factor, SP1 in SW13 cells [30]. Similarly, we found that siRNA knockdown of SP1 (Figure. 7A) reduced the level of MMP2 that was secreted by SK-MEL5+BRG1 cells (Figure. 7B). Furthermore, we detected a physical interaction between BRG1 and SP1 (Figure. 7C) and found that BRG1 was recruited to the MMP2 promoter (Figure. 7D). As was previously demonstrated in SW13 cells, BRG1 significantly increased the binding of SP1 to the MMP2 promoter [57] (Figure. 7E). This data suggests that BRG1 directly regulates MMP2 expression in melanoma cells through interactions with SP1 and by facilitating SP1 association with the MMP2 promoter. Interestingly, SP1 has been shown to preferentially interact with the BRG1 catalytic subunit in vitro [57]. Thus, a specific role for BRG1 in the activation of MMP2 and melanoma invasiveness may result from selective interactions with the SP1 transcriptional regulator.

## Discussion

Melanoma progression is a dynamic process that requires tumor cells to possess decreased adhesive interactions with surrounding cells and with the extracellular matrix at some points in the metastatic cascade and increased adhesive interactions at other times [58]. Metastatic potential also depends on adequate vascularization and the ability to degrade components of the ECM. These processes are regulated by reversible changes in the expression of genes involved in cell attachment, motility, and proteolytic degradation of the ECM [59]. Previous studies showed that SWI/SNF enzymes modulate expression of ECM related molecules in normal and cancer cells [27-30]. Furthermore, alterations in the expression of SWI/SNF components have been implicated in oncogenesis and multiple subunits have been determined to play tumor suppressive roles [60]. We previously characterized SWI/SNF subunit expression in melanoma cell lines and found that a subset of melanoma cell lines was depleted in either the BRG1 or BRM catalytic subunit. Restoration of BRG1 in a melanoma cell line that lacks BRG1 expression enhanced the expression of MITF target genes and promoted increased resistance to cisplatin [31].

To further characterize BRG1 expression in melanoma, we assayed expression in melanoma tumors. In the present study, we determined that BRG1 mRNA levels are significantly up-regulated in stage IV melanoma tumors when compared to normal skin or stage III melanoma tumors. Furthermore, primary melanoma tumors and most melanoma cell lines express high levels of BRG1 (Figure. 1B, Additional file 1) [31,32]. A recent study indicated that BRG1 expression is increased at the protein levels in primary melanoma tumors compared to dysplastic nevi, but that there is no significant difference in BRG1 levels between primary and metastatic melanoma samples [61]. However, this study found that there may be a tendency for negative to weak BRG1 expression to be associated with a better patient survival [61]. In contrast, a separate study suggested that BRG1 protein expression is frequently down-regulated in primary and metastatic melanoma compared to normal skin, but that a higher proportion of metastatic melanoma tumors express BRG1 compared to primary tumors [62]. These studies in combination with our present study suggest that BRG1 status plays a role in melanoma progression, however further investigations that utilize larger sample sizes will be required to resolve the discrepancies between the different studies.

Re-expression of BRG1 in the BRG1/BRM deficient human adrenal adenocarcinoma cell line, SW13 preferentially alters the expression of a limited number of genes that mostly encode cell surface and ECM interacting proteins [28]. Re-introduction of BRG1 in a BRG1 deficient breast cancer cell line, ALAB also had a high impact on the expression of genes that encode cell surface and ECM interacting proteins [63]. This observation and the correlation between high BRG1 levels and melanoma progression prompted us to study the impact of BRG1 on the expression of genes involved in adhesion and extracellular matrix remodeling in melanoma cells.

**Figure 8:**
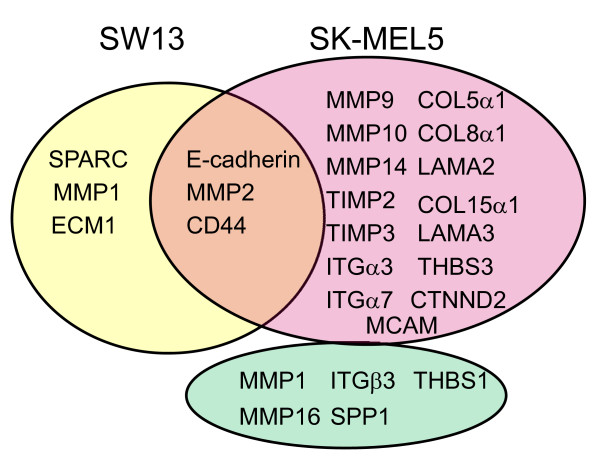
**Comparison of extracellular matrix and adhesion molecule regulation by BRG1 in SW13 and SK-MEL5 cells**. Venn diagram showing genes up-regulated by BRG1 in SW13 cells (yellow), genes up-regulated by BRG1 in SK-MEL5 cells (red), and genes down-regulated by BRG1 in SK-MEL5 cells (green). Three genes were activated by BRG1 in both SWI3 and SK-MEL5 cells (orange). Only genes that were assayed in both SK-MEL5 cells (present study) and SW13 cells [28,29,76] are shown.

Our study indicates that BRG1 activates the expression of both overlapping and distinct ECM related genes in melanoma cells as those in SW13 cells (Figure. 8). Expression of BRG1 in SK-MEL5 melanoma cells resulted in the activation of MMP2, E-cadherin, and CD44 as was also seen when BRG1 was expressed in BRG1/BRM deficient SW13 cells [28-30]. However, the expression of osteonectin (SPARC), a BRG1 dependent gene in SW13 cells, was not significantly affected by re-expression of BRG1 in SK-MEL5 cells [28] (Figure. 8, Additional File 2). Furthermore, BRG1 activated and repressed a number of cell surface and ECM interacting genes in SK-MEL5 cells that have not been identified as being BRG1 dependent in SW13 cells. Interestingly, BRG1 had opposite effects on MMP1 expression in SK-MEL5 cells compared to SW13 cells (Figure. 8) Thus, the requirement for BRG1 in the activation of specific genes is to a large extent cell context dependent. Interestingly, we found that BRG1 activated the expression of neural cell adhesion molecule (NCAM1) and δ-catenin/neural plakophilin-related armadillo protein (CTNND2), two genes whose expression is highly enriched in neural cells. Activation of these neural specific genes by BRG1 may reflect the neural crest derivation of melanoma cells.

Expression of BRG1 in melanoma cells modulated the expression of a number of ECM related genes that have opposing effects on melanoma invasiveness. In particular, BRG1 activated E-cadherin expression and down-regulated the expression of MMP1 and integrins α4 and β3. Down-regulation of E-cadherin and high levels of MMP1 and integrin αVβ3 are associated with transition from the radial non-invasive to the invasive vertical growth phase and the acquisition of metastatic potential in melanoma [37,64,65]. However, we found that BRG1 activated expression of other MMPs and integrins as well as MCAM, all of which have been shown to be important for promoting melanoma invasive ability and tumor progression [37]. Melanoma cells employ distinct strategies for invasion, each of which may differ in the degree of dependence on the different molecular regulators [6]. Interestingly, a previous study showed that dominant negative BRG1 activates integrin αV expression but still inhibits the invasive ability of fibroblasts [27]. In our studies, both a gain of function and loss of function approach indicated that BRG1 promotes melanoma invasive ability, suggesting that high levels of BRG1 promote mechanisms by which melanoma cells invade that do not rely on the induction of all known cell surface regulators.

The activation of MMP2 expression by BRG1 contributed to the increased invasive ability of BRG1 expressing SK-MEL5 cells (Figures. 5C and 5D). BRG1 was previously shown to regulate MMP2 expression in SW13 cells by a transcriptional mechanism that involves SP1 [30]. Our data indicate that BRG1 activates MMP2 expression in melanoma cells by a similar mechanism involving co-activation of SP1 mediated transcription (Figure. 7). However, BRG1 inhibited the expression of integrin β3, which is also regulated by SP1 [66]. The differential requirement for SWI/SNF function in the regulation of a transcription factor's targets has been previously observed and is not well understood [17,67]. A recent study suggests that diverse SWI/SNF complexes and sub-complexes can be recruited to different promoters and that the functional outcome of SWI/SNF activity on specific promoters may be determined by the composition of the SWI/SNF complex and the chromatin context [68]. Furthermore, the recent observation that SWI/SNF enzymes also regulate microRNA expression adds an additional layer of complexity to the overall impact made by SWI/SNF enzymes in the regulation of cellular gene expression profiles [69]. Further work will be required to decipher the mechanisms by which a high level of BRG1 results in a gene expression profile that promotes melanoma invasiveness and potentially dictates metastatic potential *in vivo*.

A number of studies have implicated SWI/SNF subunits, including BRG1, as tumor suppressors. Mutations or down-regulation of BRG1 expression occurs in multiple human tumors and haploinsufficiency of BRG1 predisposes mice to mammary tumors [70]. Furthermore, when re-expressed in SW13 cells, BRG1 interacts with the retinoblastoma protein (Rb) to induce a G1 cell cycle arrest [25]. These studies have implicated BRG1 as a tumor suppressor that curbs proliferation. In contrast, our data suggest that BRG1 expression is elevated in melanoma and promotes melanoma invasiveness. Interestingly, higher levels of BRG1 have also been associated with prostate and gastric cancer invasiveness and tumor progression [23,24]. A recent study showing that residual BRG1 expression is required for tumorigenesis to occur in INI1 deficient mice suggests that the role of BRG1 in tumorigenesis is more complex than previously thought and that the outcome of BRG1 disruption may be lineage specific [71]. We previously reported that BRG1 interacts with MITF, the master regulator of melanocyte differentiation and lineage addiction oncogene in melanoma [31]. In this study, we found that BRG1 promotes expression of NCAM1 and CTNND2, two markers that are highly expressed in neural crest derived cells. Thus, the contrasting role of BRG1 in melanoma may in part result from the lineage specific derivation of this cancer type.

## Conclusions

Our study suggests that over-expression of BRG1 contributes to melanoma progression. We have determined that BRG1 mRNA levels are higher in stage IV metastatic melanomas compared to stage III melanomas and to normal skin. Furthermore, we have determined that BRG1 modulates the expression of extracellular matrix and adhesion molecules that play an important role in melanoma metastasis. Our data indicate that modulation of extracellular matrix and adhesion molecule expression by BRG1 is associated with increased melanoma invasive ability in vitro. The down-regulation of SWI/SNF components in tumorigenesis has been elegantly demonstrated in numerous studies and is further supported by mouse models [60]. Our work adds to several other studies [23,24,72] that suggest the over-expression of a SWI/SNF component may also contribute to tumorigenesis.

## Methods

### Cell Culture

SK-MEL5 and WM-2664 melanoma cells were from the ATCC. A375SM melanoma cells were a kind gift from Dr. Menashe Bar-Eli (M.D. Anderson Cancer Center). SK-MEL5 cells expressing an empty vector or BRG1 were described in [31]. Human melanocytes were from Cascade Biologics (Portland, Oregon, USA) or Yale Cell Culture Core Facility (New Haven, Connecticut, USA). With the exception of melanocytes, all cells were grown in DMEM supplemented with 10% FBS. Human melanocytes were grown in Media 254 with added growth supplements (Cascade Biologics). The MMP2/MMP9 inhibitor, 4-Biphenylylsulfonyl)amino-N-hydroxy-3-phenylpropionamide (BiPS) was from Calbiochem (San Diego, CA, USA) and was used at 10 μM.

### RNA Isolation and Quantitative Real-time PCR

Total RNA was isolated with the Qiagen RNeasy mini kit and reverse transcribed as described [31]. Quantitative real-time PCR was performed in SYBR Green Master Mix (Qiagen, Germantown, Maryland) with an Applied Biosystems Prism 7500 PCR system and analyzed with the SDS software as described [31]. MCAM and GAPDH primers were purchased from SABiosciences (Frederick, MD, USA).

### Tumor qPCR Arrays

The Tissue Scan Melanoma qPCR Arrays (MERT501) containing cDNAs from normal skin, stage III, and stage IV melanomas were obtained from Origene Technologies (Rockville, MD, USA). The primers used to detect BRG1 (SMARCA4) were from SABiosciences (Frederick, MD, USA). BRG1 levels were normalized by amplifying with primers to Human β-actin (Forward: CAGCCATGTACGTTGCTATCCAGG) and (Reverse: AGGTCCAGACGCAGGATGGCATG). The results were averaged from values obtained by running three PCR arrays. Statistical significance was determined by utilizing a Mann-Whitney Wilcoxon test.

### Extracellular Matrix and Adhesion focused qPCR Arrays

Extracellular Matrix and Adhesion molecule RT^2 ^Profiler PCR Arrays were purchased from SABiosciences (Fedrick, MD). The primer sets in this array are described in http://www.sabiosciences.com/rt_pcr_product/HTML/PAHS-013A.html. CT values obtained for 84 extracellular matrix and adhesion molecule gene expression were normalized to a value obtained by averaging the CT values of four different housekeeping genes. For each primer set, the fold change in SK-MEL5+BRG1 cells was determined relative to values obtained in control SK-MEL5 cells +empty vector. Average values were obtained from four PCR arrays with cDNA from control cells (from three different samples) and an additional four PCR arrays with cDNA from SK-MEL5 cells +BRG1 (from three different samples). Statistical significance was calculated using the student's t test.

### Antibodies

The Tubulin antibody was from Sigma (St. Louis, Missouri, USA). FLAG M2 antibody and FLAG M2-Agarose were from Sigma. The E-cadherin, CTNND2, and MCAM antibodies were from BD Biosciences (San Jose, CA, USA), The BRG1, NCAM1 and SP1 antibodies were from Santa Cruz Biotechnology (Santa Cruz, CA, USA). The MMP2 and MMP9 antibodies were from Cell Signaling (Beverly, MA, USA). The MMP14 antibody was from Millipore (Temecula, CA, USA). The TIMP3 antibody was from Abcam (Cambridge, MA, USA). Control IgG antibody used for ChIPs was from Millipore (Billerica, MA, USA).

### Cell extracts and immunoblot analysis

Cells were lysed in 20mMTris (pH 7.4),150 mM NaCl, 2 mM EDTA, 1% Triton X, 10% glycerol, supplemented with a protease inhibitor cocktail (Sigma). SDS-PAGE and Western blotting were carried out as described [73].

### Flow Cytometry

Cells were incubated in fetal bovine calf serum (Invitrogen, Carlsbad, CA, USA) for 10 minutes at room temperature to block nonspecific antibody binding and then with the primary antibody or an isotype matched IgG control for 20 minutes at 4°C. After one wash with FACS buffer (PBS+0.5%BSA, 5% fetal calf serum, 0.1% sodium azide, cells were incubated with secondary antibody for 20 minutes at 4°C, then washed twice with FACs buffer. Cells were re-suspended in 0.1% paraformaldehyde then loaded onto a FACS-Calibur (BD Biosciences, San Jose, CA, USA). Data was analyzed using Cell Quest Pro (BD Biosciences). Statistical significance was calculated using the student's t test.

### Immunocytochemistry

Immunocytochemistry was performed as described [17] using an E-cadherin antibody (BD Biosciences) and goat anti-mouse-Alexa Fluor568 (Molecular Probes). Images were taken with a Nikon Eclipse TE2000-U fluorescence microscope at 60× magnification.

### Zymography

Zymography was performed as previously described [30]. Control SK-MEL5 and SK-MEL5+BRG1 cells were cultured in serum free medium for 36 hours. Conditioned medium was collected, normalized to cell number, and subjected to electrophoresis in a polyacrylamide gel containing 1 mg/ml gelatin. After electrophoresis, the gel was washed in 2.5% Triton X-100 for 1 hour at room temperature to remove the SDS and then incubated for 24 hours at 37°C in a buffer consisting of 5 mM CaCl_2 _and 1 μM ZnCl_2_. The gel was stained in 0.25% Coomasie Blue for 30 minutes, de-stained in methanol/acetic acid solution and photographed on a light box. Proteolytic activity was detected as white bands against a blue background.

#### siRNA Knockdowns

Acell SMART Pool siRNAs targeting BRG1 (E-010431), MMP2 (E-005959), SP1 (E-026959), and red non-targeting siRNAs (D-001960) were purchased from Dharmacon Inc. (Chicago, Il., USA) and used to transfect melanoma cells according to the manufacturer's instructions. WM-266-4 cells were transfected with control or siRNA targeting BRG1. BRG1 expressing SK-MEL5 cells were transfected with control or siRNA targeting MMP2 or SP1.

#### Adhesion Assays

Adhesion assays were performed as previously described [74]. 96 well plates were coated with laminin (10 ug/ml), collagen (type 1) (20 ugm/ml), or fibronectin (20 ugm/ml), and incubated at 4°C overnight. The plates are then washed with Wash buffer (DMEM with 0.1% BSA) and blocked in DMEM with 0.5% BSA for 45-60 minutes at 37°C. 2 × 10^4 ^cells were added to each well and incubated at 37°C for 30 minutes. Non-adherent cells were removed by washing three times with Wash buffer. The cells are then fixed with paraformaldehyde and incubated for 10-15 minutes and washed once with Wash buffer. The cells were stained with crystal violet for 10 minutes, washed with water, and dried. 2% SDS was added and the plates were incubated at room temperature for 30 minutes. Absorbance was read at 550 nm. Statistical significance was calculated using the student's t test.

#### Matrigel Invasion Assay

Invasion assays were performed using matrigel coated chambers (BD Biosciences, Bedford, MA, USA) as recommended by the manufacturer. SK-MEL5 cells expressing an empty vector or BRG1 were seeded in serum free media at a density of 1.25 × 10^5 ^cells per well on top of control or matrigel inserts. Media containing 5% FBS was used as a chemoattractant. After incubation for 16 hours, non-invading cells were removed from the upper surface and invading cells were stained with 1% Toluidine Blue and counted. Multiple fields were counted in triplicate membranes with a microscope at 20× magnification. The data shown is from two independent experiments done in triplicate. For studies involving inhibition of MMP2/MMP9, cells were pre-treated with10 μM 4-Biphenylylsulfonyl)amino-N-hydroxy-3-phenylpropionamide (BiPS) (Calbiochem, San Diego, CA, USA) for 3 hours and then plated onto the Boyden chambers in media containing 10 μM BiPS. For knockdown studies, invasion assays were performed 120 hours after transfection of control or siRNAs targeting BRG1. Statistical significance was calculated using a student's t test.

#### Co-Immunoprecipitations

Co-immunoprecipitations were performed as previously described [31].

#### Chromatin Immunoprecipitations

Chromatin Immunoprecipitations were performed as previously described [31] using FLAG to detect FLAG-BRG1 or IgG as a control. The primers used to detect the MMP2 promoter were (Forward: GGGGAAAAGAGGTGGAGAAA) and (Reverse: CGCCTGAGGAAGTCTGGAT). CD25 primers were previously described [75]. Statistical significance was calculated using the student's t test.

## Competing interests

The authors declare that they have no competing interests.

## Authors' contributions

SVS designed and performed most of the experiments. BK performed RT-PCR experiments, invasion assays, co-immunoprecipitations, and chromatin immunoprecipitations. HGM performed Westerns. HQ performed RT-PCR experiments and helped with data analysis. KVC helped design experiments and contributed resources. ILD conceived the study, designed experiments, and wrote the article. All authors approved the final version.

## Supplementary Material

Additional File 1 **Gene expression profiling of BRG1 (SMARCA4)**. The box plot is from a gene expression data set (Talantov, Clinical Cancer Research, 2005) as reported by the Oncomine microarray database. The levels of BRG1 mRNA in the indicated number (N) of malignant melanoma samples were determined to be significantly higher than those in samples from normal skin (p = 2.9 × 10^-7^). Click here for file

Additional File 2 **Extracellular Matrix and Adhesion molecule RT^2 ^Profiler PCR Array**. The expression of 84 extracellular and adhesion molecule related genes was profiled in control SK-MEL5 (empty vector) and BRG1 reconstituted SK-MEL5 cells using a focused qPCR array. For each primer set, the fold change in SK-MEL5+BRG1 cells was determined relative to values obtained in control SK-MEL5 cells +empty vector. Statistical significance was calculated using the student's t test (* indicates p < 0.05, **indicates p < 0.01). All other values were found to be not significantly different. Expression of genes that were activated by BRG1 greater than 2 fold are highlighted in red. Expression of genes that were down-regulated by BRG1 at least 2 fold are highlighted in green. N.D. represents genes that were not detected by the qRT-PCR assay or that had CT values > 34. Click here for file

Additional File 3 **Cell Confluency and SWI/SNF Subunit Expression**. Western blot showing the effect of increasing confluency on the expression of BRG1, BRM, and INI1 in WM2664 cells that were cultured in the absence of serum. Tubulin is a loading control. Click here for file
