# Supplementary figures and images for: Modulation of extracellular matrix/adhesion molecule expression by BRG1 is associated with increased melanoma invasiveness
Source: Mol Cancer. 2010 Oct 22;9:280. doi: 10.1186/1476-4598-9-280 (PMC3098014; doi:10.1186/1476-4598-9-280)

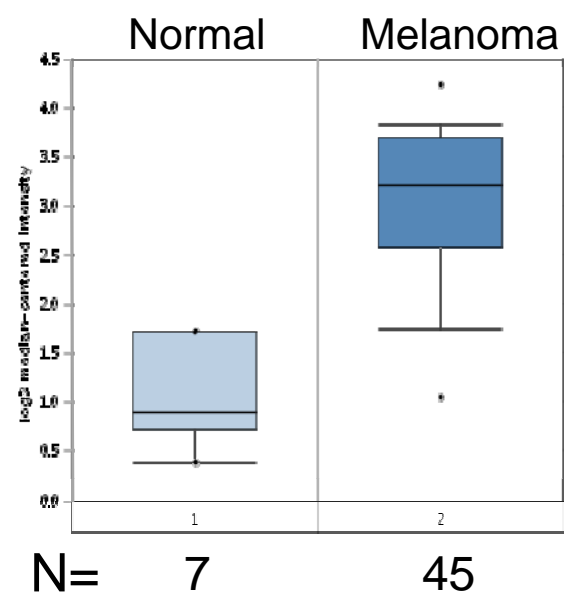

Supplement: Additional File 1 — Gene expression profiling of BRG1 (SMARCA4). The box plot is from a gene expression data set (Talantov, Clinical Cancer Research, 2005) as reported by the Oncomine microarray database. The levels of BRG1 mRNA in the indicated number (N) of malignant melanoma samples were determined to be significantly higher than those in samples from normal skin (p = 2.9 × 10^-7^). [file 1476-4598-9-280-S1.PDF]

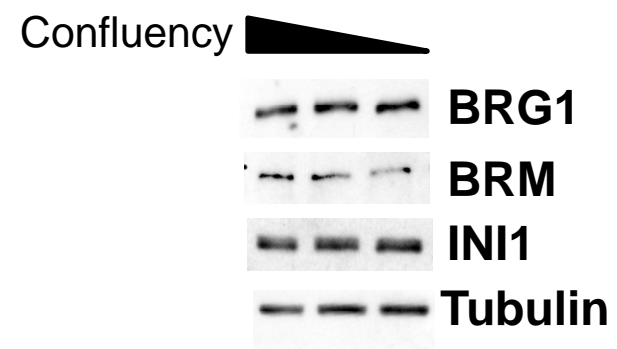

Supplement: Additional File 3 — Cell Confluency and SWI/SNF Subunit Expression. Western blot showing the effect of increasing confluency on the expression of BRG1, BRM, and INI1 in WM2664 cells that were cultured in the absence of serum. Tubulin is a loading control. [file 1476-4598-9-280-S3.PDF]
